# Two‐Way Gateway Designs to Allow Free Movement Between Safe Havens for Bettongs: A Captive Trial

**DOI:** 10.1002/ece3.71481

**Published:** 2025-05-22

**Authors:** Xin Lei Pan, Julia M. Hoy, Megan J. Brady, Adrian D. Manning, Megan C. Edwards

**Affiliations:** ^1^ School of Agriculture and Environmental Science University of Southern Queensland Darling Heights Queensland Australia; ^2^ Hidden Vale Research Station Turner Family Foundation Grandchester Queensland Australia; ^3^ School of the Environment The University of Queensland Brisbane Queensland Australia; ^4^ The Fenner School of Environment and Society Australian National University Canberra Australian Capital Territory Australia

**Keywords:** animal behaviour, captive wildlife, conservation fencing, mammals, microchip‐automation, safe haven

## Abstract

Introduced predators in Australia are one of the major causes of native fauna species decline, with attempts to address this decline including predator control, wildlife reintroductions and predator‐proof conservation fencing. The efficacy of conservation fencing means this tool is increasingly used to counteract species decline; however, there is growing awareness that fences can also contribute to issues such as overpopulation, prey naivety and restrictions to natural dispersal and genetic diversity. This research aimed to investigate the potential for two‐way gateways within fences to help address these limitations, allowing movement of native wildlife while reducing introduced predators. Rufous bettongs ( *Aepyprymnus rufescens* ) were used for this research as a model species representing ‘critical weight range’ mammals. Seven individually housed captive rufous bettongs were used to investigate interactions with and preference for five gateway designs. Using adaptive methodology, individual rufous bettongs were presented with four of the five gateways and their responses were analysed. The seven rufous bettongs at different life stages and sizes were all able to use all five gateway designs presented to them and showed a preference for designs made from PVC pipe. Gateway positions also significantly influenced the frequency of interactions with the gateways, with bettongs showing a preference for gateways along the edge of the fence rather than the middle. The results from this study are an important step in the development of innovative strategies for safe haven design and improving the performance of semi‐permeable fenced areas for conservation of species impacted by introduced predators. Further testing of these gateways in situ will contribute to the field of ‘coexistence conservation’ – the long‐term, iterative and adaptive process to enable the coexistence of threatened species and native or introduced predators.

## Graphical abstract

Rufous bettongs were provided with a variety of gateway designs to allow movement of native wildlife between fenced reserves. Bettongs successfully learned to use all gateway designs, showing potential for future in situ conservation safe havens.

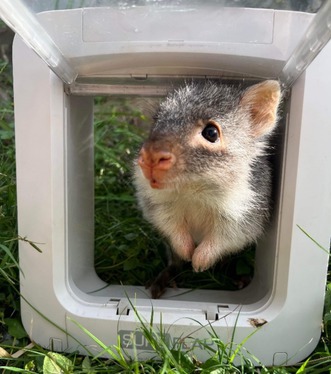

## 1 Introduction

Introduced predators such as feral cats ( *Felis catus* ) and red foxes ( *Vulpes vulpes* ) have negatively impacted Australian native flora and fauna (Woinarski et al. 2015; Legge et al. 2017; Jessop et al. 2021; Stobo‐Wilson et al. 2022). Cats alone (domestic, feral and unowned) are responsible for the estimated loss of 459 million native Australian mammals annually (Murphy et al. 2019). A diverse combination of strategies has been conceptualised and trialled by practitioners over the past decade to mitigate predation impacts or enable recovery of native fauna populations (Short and Hide 2014, 2015; Ruykys and Carter 2019; Roshier et al. 2020).

Conservation translocations are one such method, primarily aiming to return native species to their former range where they no longer persist, or to replenish/restock an existing declining population (Seddon et al. 2007; IUCN/SSC 2013; Sutton and Lopez 2014). However, the presence of introduced predators has been established as a leading cause of reintroduction failures in Australia (Short 2009; Moseby et al. 2011; Sheean et al. 2012; Batson et al. 2015; Morris et al. 2021).

Conservation translocations into predator‐proof fenced reserves (often called ‘safe havens’) have shown greater success in establishing wildlife populations than those outside of fenced reserves (Moro 2003; Dickman 2012; Woinarski et al. 2015; Legge et al. 2018). Safe havens have made it possible for native prey species such as greater bilbies ( *Macrotis lagotis* ) (Moseby and O'Donnell 2003), burrowing bettongs ( *Bettongia lesueur* ) (Moseby et al. 2018), eastern bettongs ( *Bettongia gaimardi* ) (Batson et al. 2016) and eastern quolls ( *Dasyurus viverrinus* ) (Wilson et al. 2021) to increase their populations in the absence of introduced predators.

However, wildlife populations within safe havens face restrictions to natural dispersal (potentially leading to overabundance that can cause ecosystem imbalance) (Moseby et al. 2018), limited gene flow (that can reduce genetic diversity) (Lott et al. 2020) and prey naivety (failure of prey to recognise threat of introduced predators) (Jolly et al. 2018; Harrison et al. 2023; Read et al. 2023). In response to some of these limitations, one‐way gateways have been developed to allow free movement (i.e., dispersal) out of reserves (Crisp and Moseby 2010; Butler et al. 2019; Moyses et al. 2020). Although the designs were successful with some species and allowed for dispersal outside of fenced areas, one‐way designs do not address limited genetic diversity or prey naivety within fenced reserves.

Two‐way gateways allowing the free movement of native species in and out of fenced reserves could contribute to improved genetic diversity, by allowing animals to seek mates from both within and outside the reserves. Also, two‐way gateways have the potential to reduce prey naivety through achievement of the ‘Goldilocks Zone of Predation’, that is, low enough predation levels that a native species does not become extinct, but high enough predation to reduce naivety and drive adaptation (Evans et al. 2021). Previous studies have shown that low predation pressure can reduce prey naivety in native species (Moseby et al. 2019; Evans et al. 2021). Two‐way gateways that allow some predator access (but not all) could assist in reducing prey naivety through facilitating low predation pressure in fenced reserves and immediately outside, rather than complete isolation from introduced predators.

It is, however, crucial for two‐way gateways to appeal to target native species and discourage (although potentially not exclude all) introduced predator use. A variety of gateway designs could be used to achieve this, including the use of deterrents or through the use of gateways that only allow certain individuals access.

Visual deterrents such as motion‐triggered flashing lights (e.g., Foxlights) have been used to deter predators such as Andean foxes ( *Lycalopex culpaeus* ), red foxes, common leopards ( *Panthera pardus* ) and pumas ( *Puma concolor* ) from taking livestock (Zarco‐González and Monroy‐Vilchis 2014; Ohrens et al. 2019; Naha et al. 2020; Hall and Fleming 2021). Flashing lights were found to be more efficient than a constant light source in deterring felids than canids (Ohrens et al. 2019).

Microchip‐automated gateways are similar to a traditional pet door, except they only allow use by the microchipped, registered animal (Muns et al. 2018; Edwards et al. 2020), thereby excluding introduced predators (or other non‐target animals). The use of microchip‐automated doors has been successfully tested on a variety of native species such as common brushtail possums ( *Trichosurus vulpecula* ) (Watson et al. 2021), brush‐tailed phascogale ( *Phascogale tapoatafa* ) (Edwards et al. 2019; Watson et al. 2022), northern brown bandicoots ( *Isoodon macrourus* ) (Edwards et al. 2020) and a bridled nail‐tail wallaby (Muns et al. 2018). Such designs could improve conservation translocation successes and better facilitate re‐establishment of species to their former ranges with minimal human management post release.

Rufous bettongs ( *Aepyprymnus rufescens* ) are nocturnal, mostly solitary, potoroid marsupials that inhabit grassy woodlands of eastern Australia (Dennis 2023). Rufous bettongs are considered ‘Least Concern’ by the IUCN; however, population declines have been observed within the southern parts of their range (Dennis 2023). Rufous bettongs are within the Critical Weight Range (CWR; 35–5500 g) (Burbidge and McKenzie 1989), weighing 1.3–3 kg (Dennis 2023) and due to their abundance are a suitable model research species for studies related to improving conservation strategies for Australian CWR mammals.

The aim of this study was to investigate the potential for five different two‐way gateway designs that are small enough to exclude some introduced predators but allow free movement of CWR mammals through a fence, using captive rufous bettongs as a model species.

Specifically, this study examined:

- Whether rufous bettongs at different life stages and sizes would use the different gateway designs.
- The duration for rufous bettongs to successfully learn to use the different gateway designs.
- Whether rufous bettongs displayed a preference for a particular gateway design.

## 2 Materials and Methods

### 2.1 Study Animals and Housing

**FIGURE 1:**
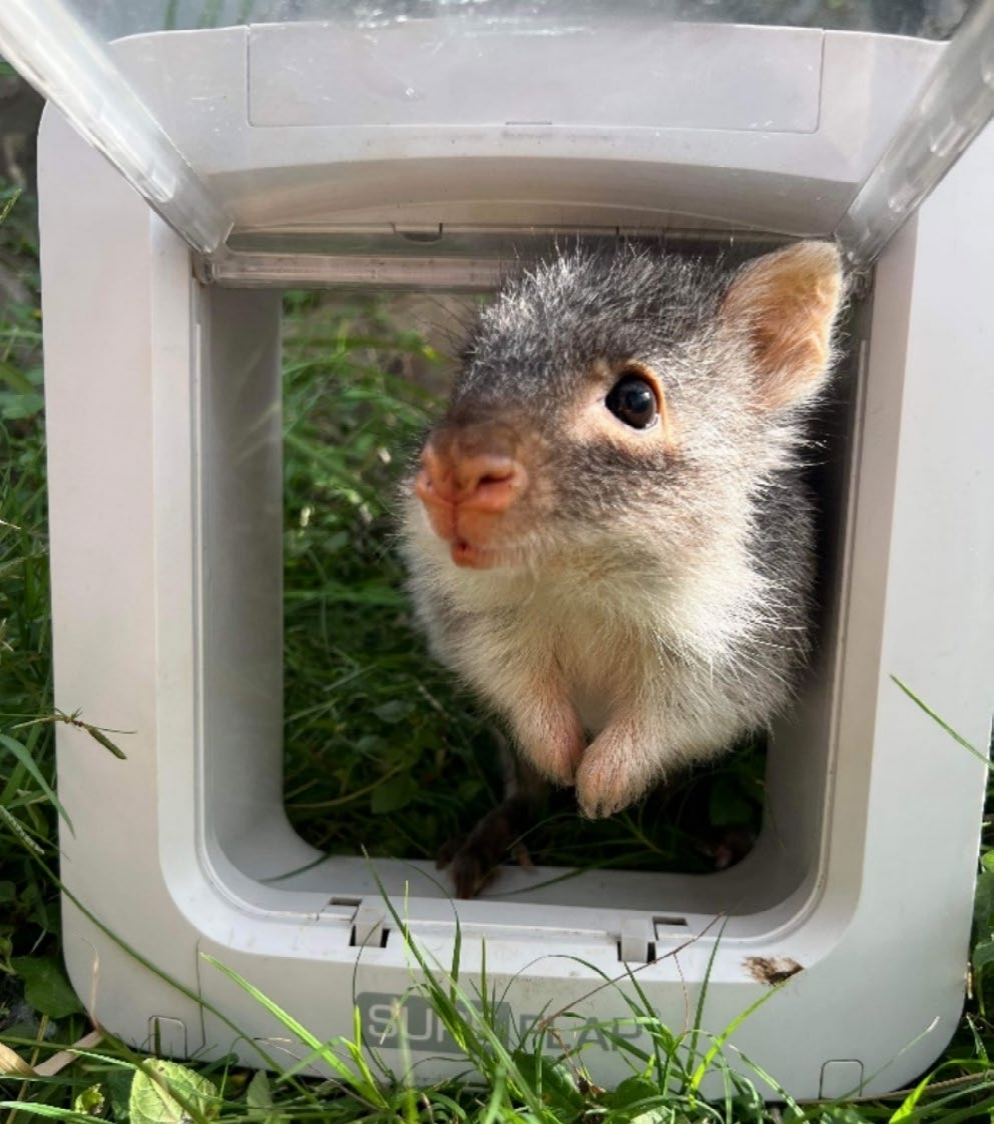
Rufous bettong looking through a microchip‐automated gateway.

Seven captive‐bred rufous bettongs (hereafter bettong) (Figure 1), both male and female of a range of ages (6 months—7 years), housed at the Hidden Vale Research Station (located west of Brisbane in south‐east Queensland) were included in this study. All bettongs have been microchipped with individual PIT tags. Six of the seven bettongs were related (parent and offspring) and one was hand raised (due to complications after pouch emergence). The bettongs were housed individually in pens (12 m × 6 m × 7 m) to ensure independent decision‐making. All pens were designed and furnished similarly with native flora, logs, rocks and tussock grass to minimise variability between pen designs.

Individuals were moved into their respective pens between 3 and 4 weeks prior to testing, for them to habituate to their environment. Food intake and weights of individuals were monitored throughout to ensure that there were no negative impacts on the bettongs throughout the testing. Their standard diet in captivity consisted of oats, dog kibble, mealworms, egg (twice weekly), macropod pellets/lucerne chaff and a daily mixture of fresh produce: three types of vegetable (70%) and two types of fruit (30%). Food provided to the bettongs was the same across all individuals on the same night, to avoid variation in food‐related behaviours.

### 2.2 Gateway Designs

**FIGURE 2:**
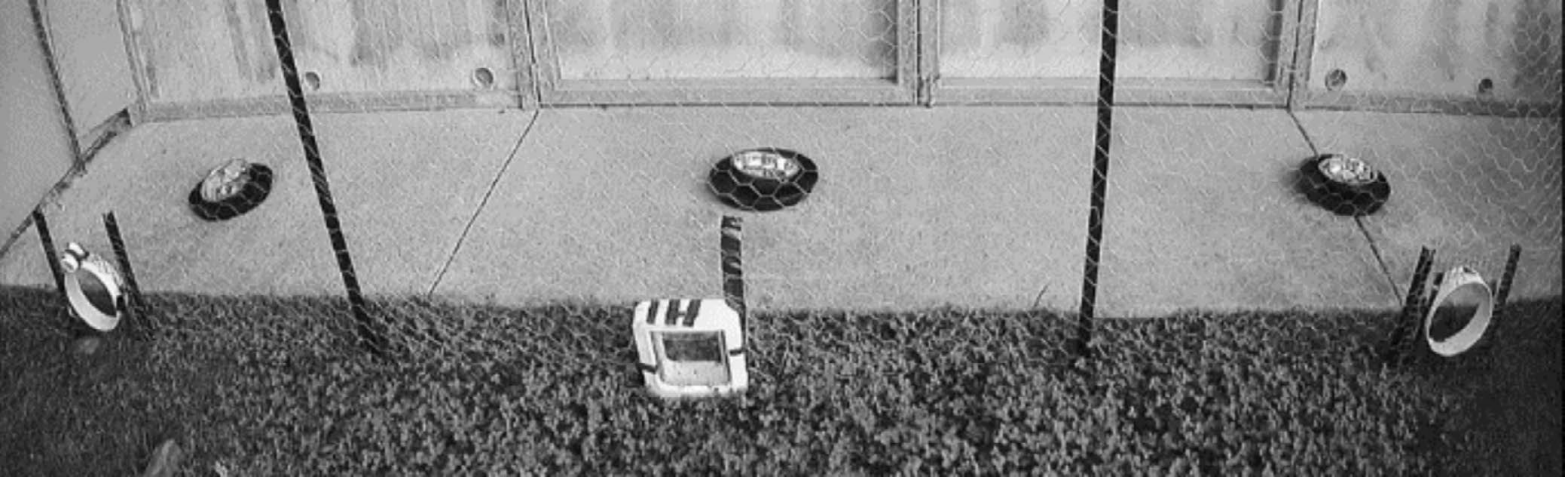
Standard food placements used as attractants to encourage gateway usage. Three positions for gateways attached to the fence. Example of randomised sequence: Left—250‐light gateway, middle—microchip‐automated gateway; right—250‐pipe gateway.

A fence was constructed in each pen to separate the area where food was provided from the rest of the pen (Figure 2). Five gateway designs were used:

- PVC pipe gateways in two sizes (160 mm and 250 mm diameter) are a simple, efficient to install gateway that is permeable to both the target species and some predators;
- The same sized pipe gateways with a motion activated light (Lytworx Motion Sensor Outdoor Ball Light, Bunnings, Australia) attached to potentially deter predators from passing through the gate; and
- A microchip‐automated gateway (SureFlap Microchip Pet Door, Cambridge, England) that allows only the intended microchipped animal to pass through and excludes all non‐target animals.

Each bettong's food was divided into three equal portions and positioned evenly spaced on the concrete behind each gateway to encourage usage (Figure 1).

Individual bettongs were presented with four of the five gateway designs using adaptive methodology (Table 1). The seven bettongs were split into two rounds of tests (Table 1), with three bettongs in Round 1 (May—June 2022) and four bettongs in Round 2 (July—September 2022).

**TABLE 1:** Gateway designs presented to bettongs in each round.

Round 1	Round 2
250‐pipe gateway	160‐pipe gateway
250‐light gateway	160‐light gateway
Microchip‐automated gateway	Microchip‐automated gateway
160‐pipe gateway	250‐pipe gateway

*Note:* Round 1 = May–June 2022. Round 2 = July–September 2022.

### 2.3 Habituation to Individual Gateway Designs

Each bettong was exposed to one gateway design at a time, in a randomised order. The position of the gateway introduced for each habituation period remained consistent across all bettongs at the same time, with a different gateway design presented in that position to each bettong. The pipe and light gateways were introduced to the bettongs for seven nights each. The microchip‐automated gateways were introduced in four stages (minimum of two nights per stage—minimum eight nights): (1) fully opened, (2) half opened, (3) fully closed and (4) locked and functional, based on previous studies involving similar methods (Edwards et al. 2019, 2020; Watson et al. 2021, 2022). The bettongs progressed to the next stage during the microchip‐automated gateway habituation when they successfully used the gateway for two nights without applying other means, such as digging under the fence, to move between the concrete area and the rest of the pen. A one‐week break followed the last night of habituation for all bettongs to avoid possible bias during the preference test, such as the position of the last gateway presented influencing their use.

### 2.4 Preference Test

During the preference test, the three gateway designs were presented simultaneously in a multiple stimulus experiment (Martin et al. 2013), in three randomised sequences for each bettong for two nights per sequence. This allowed the three gateway designs to be cycled through all possible positions on the fence, investigating whether gateway interactions were a result of design or position on the fence. The preferred gateway or position was considered the most frequently used gateway or position (Martin et al. 2013).

### 2.5 Gateway Size Test

At the conclusion of the preference test, a gateway size test was done to determine the appropriate diameter for the pipe and light gateways. The two sizes were presented simultaneously to the bettongs for a period of 3 days. The first diameter chosen was 250 mm (250‐pipe gateway) and the second diameter was 160 mm (160‐pipe gateway). The 250‐pipe gateway was chosen based on the smallest size suitable for the largest measurement taken of a bettong (female with large pouch young) and the 160‐pipe gateway was chosen because it may be more likely to deter foxes' and large feral cats' usage when implemented in the field.

### 2.6 Behavioural Activity and Analyses

Video footage was recorded for 11 h each night from 1700 to 0400, based on previous evidence that the bettongs in this facility showed no activity outside those hours. Existing pen surveillance cameras provided a limited view of the fence and gateways due to permanent structures in the pens and distance from the fence. Therefore, two additional infrared security cameras (Swann SWDVK‐845808 V) were installed in each pen from angles closer to the fence to observe individual bettong behaviours around the gateways. All occurrence sampling was done to document four main gateway interactions: partial entry, partial exit, full entry and full exit (Table 2).

**TABLE 2:** List of main gateway interactions based on behaviours observed using description adapted from Hoy (2010).

Gateway interaction	Description
Full entry	Complete entry on to the concrete area: where the entire body passes through the gateway from the main pen area on to the concrete area
Full exit	Complete exit from the concreted area: where the entire body passes through the gateway from a concrete area out to the main pen area
Partial entry	Incomplete entry on to the concrete area: where only part of the body passes through the gateway (i.e., head and/or limbs) to enter the concrete area
Partial exit	Incomplete exit from the concreted area: where only part of the body passes through the gateway (i.e., head and/or limbs) to leave the concrete area

All analyses were conducted using R version 4.2.1 (R Core Team 2022). General linear models and ANOVAs were used to test the effects of gateway design and gateway position, allowing for the effect of each ‘Bettong’ trialcombination. Analyses were carried out on the numbers of partial, full and combined interactions with the different gateway designs. Least‐squares means, along with standard errors and 95% confidence limits, were produced for each gateway design and each gateway position to assess the significance of design and position on the frequency of rufous bettong and gateway interactions. Tukey post hoc tests were then performed to compare differences between each of the gateway designs and gateway positions. A statistical significance level of *p* < 0.05 was used for all the tests. Due to the small sample size of this study, descriptive statistics and behavioural observations were also used to discuss any other results.

## 3 Results

The seven bettongs were all able to use all five gateway designs presented to them. The pipe gateways had significantly higher full interactions (bettongs passed all the way through the gateway, as per Table 2) (160 mm with light: *p* = 0.0101; 160 mm without light: *p* = 0.0163; 250 mm with light: *p* = 0.0003; and 250 mm without light: *p* = 0.0006) than the microchip‐automated gateways of their respective rounds of preference tests. The total count of full interactions with the 250‐pipe gateway was significantly higher (*p* = 0.0042) than with the 160‐pipe gateways in the gateway size test.

### 3.1 Habituation Period

All seven bettongs passed through the respective gateways presented on the first night of habituation. The shortest time between first sighting on camera and first movement through a gateway on the first night was just under 30 s (250‐light) and the longest time was just over an hour and a half (160‐light).

Time taken for the first complete movement through a gateway was the longest on the first night of gateway presentation for the majority of the bettongs. However, shorter durations were recorded for the first usage of successive gateway designs presented. During the microchip‐automated gateway habituation, more than half of the bettongs were easily able to pass each learning stage within the planned two nights, whereas two bettongs each required extra night(s) during the fully closed and fully opened stages, respectively. One bettong was able to complete the microchip‐automated gateway habituation despite the door accidentally closing fully (rather than being held open slightly) during stage two (half open → fully closed), resulting in completion two nights earlier than her conspecifics.

### 3.2 Preference Test

**FIGURE 3:**
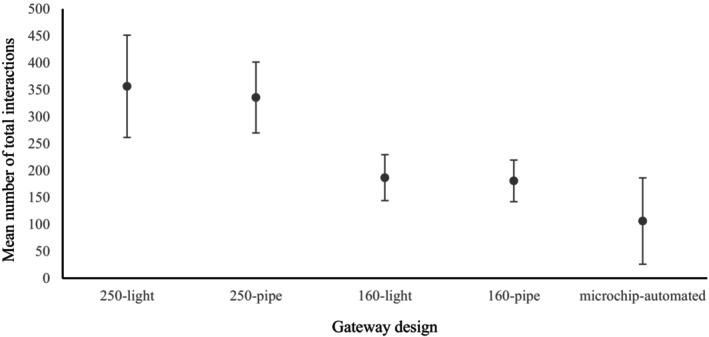
Mean number of all gateway interactions (±SE) of rufous bettongs during the preference tests.

When all interactions in the preference tests were analysed (i.e., all full and partial interactions combined), the light and pipe gateways both had a higher frequency of interaction than the microchip‐automated gateway for all bettongs in both rounds of preference tests (Figure 3).

**FIGURE 4:**
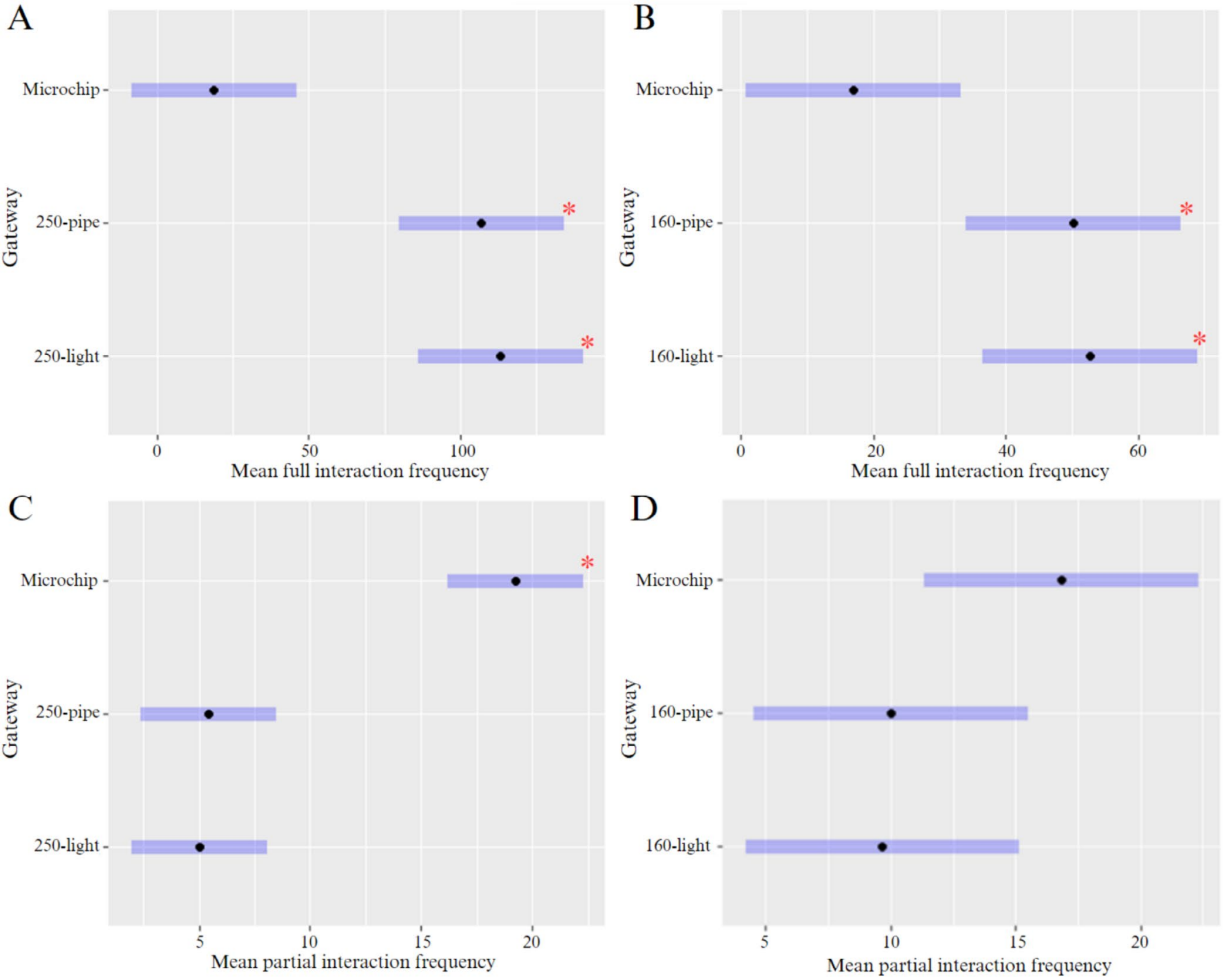
Mean interactions (95% ±CI) of all bettongs with different gateway designs for preference test Round 1 (A and C) and Round 2 (B and D), including full interactions (A and B) and partial interactions (C and D). *indicates significant variation between designs (*p* ≤ 0.05).

The 250‐light and 250‐pipe gateway designs of Round 1 had significantly higher (*p* = 0.0003 and *p* = 0.0006, respectively) full interactions by bettongs than the microchip‐automated gateway (Figure 4). Similarly, bettongs had significantly more full interactions with the 160‐light and 160‐pipe gateways (*p* = 0.0101 and *p* = 0.0163, respectively) than the microchip‐automated gateway in Round 2 (Figure 4). There was no significant difference in the mean frequency of full or partial interactions between light and pipe gateways in their respective test rounds. Partial interactions were significantly higher with the microchip‐automated gateway (*p* < 0.0001) in Round 1, and no significant variation was found for partial interactions between designs in Round 2 (Figure 4).

**FIGURE 5:**
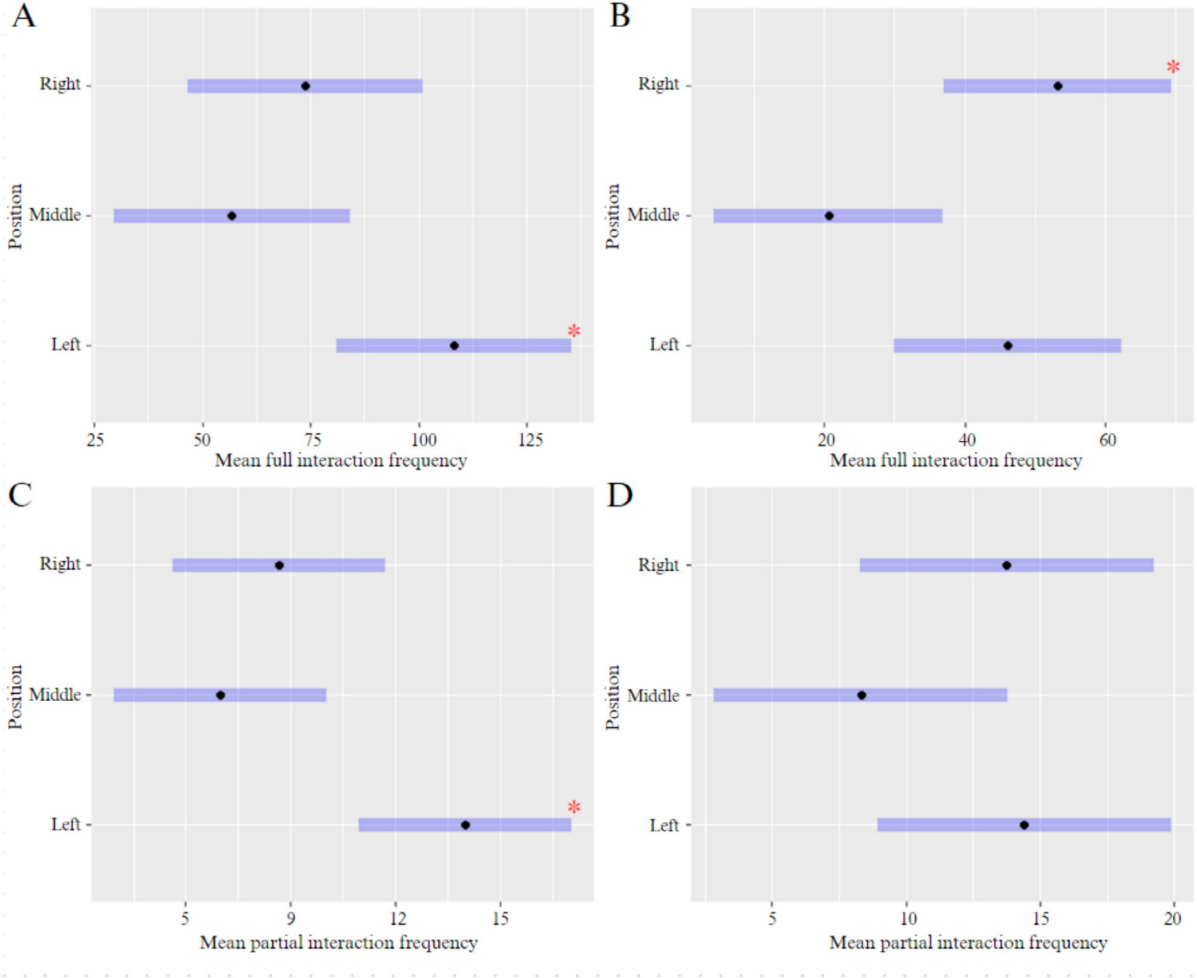
Mean gateway interactions (95% ±CI) of all bettongs for preference tests Round 1 (A and C) and Round 2 (B and D) with respect to position on the fence, including full interactions (A and B) and partial interactions (C and D). *indicates significant variation between position (*p* ≤ 0.05).

The middle position had significantly fewer full and partial interactions (*p* = 0.0320, *p* = 0.0096 and *p* = 0.0467) by bettongs in Round 1 than the other positions (Figure 5). Bettongs in Round 2 also had significantly fewer full interactions (*p* = 0.216) with gateways positioned in the middle.

### 3.3 Gateway Size Test

**FIGURE 6:**
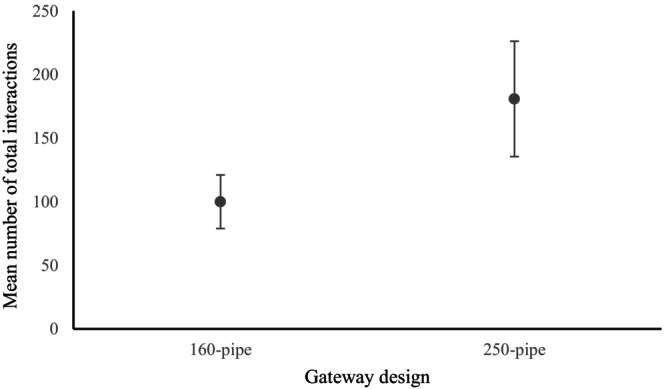
Mean number of total gateway interactions (±SE) of all bettongs during the gateway size tests.

There were significantly more full interactions with the 250‐pipe gateway (*p* = 0.0042) than with the 160‐pipe gateway (Figure 6), there were significantly more partial interactions with the 160‐pipe gateway (*p* = 0.0331) than with the 250‐pipe gateway. The position of the gateways was only significant for partial interactions (*p* = 0.0371), with bettongs interacting with the left more than with the middle or right.

## 4 Discussion

All gateways were used by all bettongs, with a preference (more frequent use) for the pipe gateway design (with or without the light) over the microchip‐automated gateway. These gateways therefore show potential for safe haven projects and the use of these gateways in situ.

### 4.1 Habituation to Gateway Designs

Regardless of their age or size, all seven bettongs showed an ability to learn and use each of the gateways presented, as most movements through the gateways occurred in less than an hour on the first night that each gateway design was introduced. When exposed to subsequent gateway designs, there was a substantial reduction of learning durations recorded for five of the seven bettongs. This could indicate that while it may take some time for the bettongs to move through the first gateway, they can subsequently adapt to new designs. One of the bettongs was able to complete the microchip‐automated gateway habituation phase quicker than others, similar to the results of a study with bandicoots that indicated the potential for conspecifics to achieve similar proficiency at using microchip‐automated doors when put on different habituation timelines (Edwards et al. 2020). These results indicate that there is potential for the duration of habituation periods used in this study to be shortened for future training in captivity. Similarly, bettongs (and potentially other CWR species) may choose to use the gateways in fenced reserves in situ relatively quickly, though there may be differences between captive and wild animals that require further investigation (Crates et al. 2022).

### 4.2 Preference of Gateway Designs

The bettongs showed a significant preference for the pipe gateway (with and without lights) compared to the microchip‐automated gateway. Microchip‐automated gateways required the bettongs to learn when the locking mechanism disarmed and to perform an additional action of pushing a plastic flap to pass through. These additional requirements may reduce the appeal of gateway usability (Butler et al. 2019; Coates 2013), especially in situations where bettongs seek the most efficient point of entry or exit. While it may take longer for bettongs to habituate to full use of the microchip‐automated gateways, these gateways have the advantage that they may exclude all introduced predators, and so may be preferred over other designs by land managers if that is the desired outcome. Testing on long‐term use in the field would be required to ensure longevity and the ability to withstand outdoor conditions for extended periods.

There was no significant difference between the frequency of interactions between the pipe gateway with and without lights despite some individuals using one more than the other. However, it is important to note that there may be differences with wild bettongs (and other species) as they may develop different behaviours and phenotypic traits from those bred and raised in captivity (Crates et al. 2022), where artificial light is common. Light deterrents have been trialled on predator species around the world (Zarco‐González and Monroy‐Vilchis 2014; Ohrens et al. 2019; Naha et al. 2020; Hall and Fleming 2021), but not in Australia. Testing light deterrents with a variety of predators in Australia, as well as positioning of the light deterrents and incorporating other stimuli (auditory/olfactory deterrents) to the design, to avoid rapid habituation of deterrents (Zarco‐González and Monroy‐Vilchis 2014) should be investigated as a method of deterring predators from using pipe gateways.

Gateways positioned in the middle of the fence had fewer interactions from bettongs than the left and right positions. This may be influenced by a possible funnel effect as seen in other research (Butler et al. 2019), because the bettongs in this study followed the walls of the pens towards the gateways in the left and right positions, and also because the gateways in the left and right positions were along the usual path that the bettongs travelled on a regular basis. This is an indication that apart from ensuring that the gateway designs appeal to target species, field surveys to identify suitable habitat and signs of existing populations (tracks and/or scats) before implementing safe havens and installing gateways may help to increase the chances of wild conspecifics encountering the gateways, thereby increasing the chances of them using the gateways.

A significant preference for the larger 250‐pipe gateways was established across the seven bettongs of the study. The 250‐pipe gateway was large enough to provide individual bettongs with more locomotion options to move through it, and they could bring their whole body through in one motion if desired. The 160‐pipe gateway, however, was too small for them to bring their whole bodies through in one motion and mostly limited their locomotion option to saltation (slow walk (Johnson 1980)), where they had to put their front half (forelimbs and head) through first, then pull their back half (hind limbs simultaneously) through.

The position effect was not observed in the gateway size test, which may imply that when the bettongs had a strong preference for the 250‐pipe gateway, they were able to seek out that specific design regardless of its position on the fence. Butler et al. (2019) found a preference by burrowing bettongs for one‐way gateways located in corners rather than on straight sections of fences (funnel effect). However, only placing gateways in corners significantly limits the number of available positions for gateways, thereby limiting the chances of gateway encounter and usage. Therefore, as the bettongs from this study were able to overcome position effects when they have a strong preference for a specific design, this preference could be expressed by wild conspecifics in field trials, such that gateways along straight sections may be used as frequently as those in corners.

The results of this study were derived from data collected on captive animals, and as such, while the results may help inform the use of these gateways in safe havens in situ as proof of concept, there are likely to be differences between captive and wild animals (Crates et al. 2022). Field trials are required with wild individuals to determine differences in their temperament and use of the gateways. These tests in captivity also serve as an indication of the short‐term durability of the gateway materials and designs when exposed to bettongs and the weather. This area requires further testing to determine the level of management required for gateway maintenance when they are established in situ.

The attractant (daily food provisions) used may have increased the frequency of gateway usage. Therefore, further testing to assess behaviours and learning abilities of bettongs when not provided attractants or exposed to different attractants that simulate long‐term field conditions is required. Variation of habitat attributes around gateways could also influence gateway usage in situ. Dense vegetation that resembled levels of cover used by target species to avoid predation could be trialled in future in situ studies. The sample size for this study was necessarily small due to the limited availability of animals, reflecting the practical challenges of working with wildlife in conservation‐focused research. While we acknowledge that this small sample size may limit the generalisability of our findings, we demonstrate that bettongs are able to use a variety of gateway designs to access a food reward. Despite the constraint of small sample size, the trial yielded valuable observations on bettong behaviour and provided countless useful insights for refinement of gateway designs, such that the gateways have since been used in in situ trials in safe havens (Brady et al., unpublished data).

### 4.3 Future Research Directions

Depending on the goal of the safe haven, the use of pipe and/or microchip‐automated gateways could provide land managers with the ability to allow fenced areas to be semi‐permeable. Microchip‐automated gateways have the potential to be highly effective at preventing introduced predators from infiltrating a safe haven since they only permit usage by target animals with a recognised microchip. In particular, microchip‐automated gateways could play an important role in translocation projects where founders or young are pre‐conditioned in captivity prior to release, or areas where intensive trapping occurs allowing a critical mass of the wild population to be microchipped. It may be worthwhile exploring the possibilities of round microchip doors and automated designs that open/close without having animals push through them, since animals may be more inclined to use a gateway if they are able to move through the structure using their natural locomotion behaviours (Butler et al. 2019; Coates 2013). These design changes could be the alterations needed to increase microchip‐automated gateway usage. The use of artificial intelligence (AI) technologies has been explored in other fields such as to monitor individual health of farmed cattle (Periyanayagi et al. 2022) and distinguish tagged versus untagged vultures (Santangeli et al. 2022). Hence, exploring the capabilities of programming or teaching AI to distinguish target species from introduced predators using facial recognition technology and developing AI models capable of being trained to identify multiple target species (Congdon et al. 2022) should also be explored so that future generations in situ may also use the gateways without a microchip being required.

### 4.4 Project Significance

This research assists in creating semi‐permeable safe havens that allow the movement of free‐living wildlife between fenced and unfenced areas. These could be traditional safe havens (large, fenced areas that are generally maintained to be impermeable to introduced predators; *sensu* Legge et al. 2018), or clusters of smaller “mini‐safe havens” that are permeable to the target species and allow for in situ adaptation to the key threats that lead to extirpation (Smith et al. 2023). The gateways used in this research could be implemented in safe havens and mini‐safe havens in a variety of landscapes to promote genetic mixing, natural dispersal and reduce prey naivety, as they allow free movement of target species through fences, while potentially excluding some predators. This contributes to the theme of ‘coexistence conservation’, whereby conservation focus and efforts gradually progress from eliminating or excluding introduced predators, to assisting target species in learning and adapting to their presence (Evans et al. 2022). These semi‐permeable gateways represent one part of the solution to this complex issue.

## Author Contributions

**Xin Lei Pan:** conceptualization (equal), data curation (lead), formal analysis (lead), writing – original draft (lead), writing – review and editing (supporting). **Julia M. Hoy:** conceptualization (equal), resources (equal), supervision (equal), writing – review and editing (supporting). **Megan J. Brady:** conceptualization (equal), resources (equal), supervision (equal), writing – review and editing (supporting). **Adrian D. Manning:** conceptualization (equal), supervision (equal), writing – review and editing (supporting). **Megan C. Edwards:** conceptualization (equal), project administration (lead), resources (equal), supervision (equal), writing – original draft (supporting), writing – review and editing (lead).

## Conflicts of Interest

The authors declare no conflicts of interest.

## Data Availability

Data are available at https://research.usq.edu.au/item/zwzy9/two‐way‐gateways‐for‐bettongs.
